# How to prevent the next Marathon Pharmaceuticals

**DOI:** 10.12688/f1000research.13678.1

**Published:** 2018-01-17

**Authors:** Frank S. David, Richa Dixit

**Affiliations:** 1 Pharmagellan LLC, Milton, MA, , 02186, USA

**Keywords:** FDA, orphan, pharmaceutical, price, regulation

## Abstract

In recent years, several drug companies have exploited U.S. regulatory policies to acquire exclusive rights to cheap therapies and substantially raise their prices, and Federal agencies and state governments are exploring various ways to prevent or punish such behavior in the future. Among these cases, however, Marathon Pharmaceuticals’ handling of Emflaza (deflazacort) is unique, because the drug was previously only available abroad, and was never previously sold in the U.S. before the company obtained FDA approval for it. Thus, laws and policies designed to address price hikes on already-marketed drugs are unlikely to prevent additional Marathon-like scenarios. In this article, we describe in more detail the unique features of Emflaza compared with these other recent cases of drug price increases, determine the likelihood that similar situations will arise in the future, and explore legislative and administrative options to specifically prevent such behavior.

## Introduction

When Marathon Pharmaceuticals obtained U.S. approval for Emflaza (deflazacort) in Duchenne’s muscular dystrophy (DMD) in 2017, legislators and industry critics vilified the company’s plan to price the drug at $89,000 per year – not for the high cost *per se*, but for allegedly taking unfair advantage of U.S. regulatory and pricing policies to profit handsomely from a decades-old drug available abroad for under $5 a day.

Although Marathon gained the public’s attention in the wake of several other high-profile cases of alleged drug pricing misconduct, this case is actually unique. Unlike firms that have acquired U.S.-marketed agents and then hiked their prices, Marathon gained FDA approval of a drug that was already commercialized abroad, but not in the U.S., then set a U.S. price far higher than the international one. This distinction does not automatically exonerate Marathon, but it highlights a special challenge for regulators and policy-makers seeking to balance access with affordability. It also suggests that there may not be a “one size fits all” approach to punishing and/or preventing companies that engage in the broad range of improper drug pricing behaviors that have been seen to date.

The details of the Marathon case raise three questions. First, what specific aspects of the company’s behavior were inappropriate, and distinguish Marathon from other companies that launch high-priced drugs in the U.S.? Second, how prevalent and important are future scenarios like Emflaza likely to be? And finally, what legislative, administrative, and non-policy approaches might effectively prevent and/or punish similar activities?

## Distinguishing Emflaza from “appropriate” orphan drugs

Deflazacort, a synthetic corticosteroid originally developed by Merrell Dow Pharmaceuticals, has been available since the 1990s outside the U.S. In 1996, preliminary data in 196 DMD patients showed it was comparably effective to prednisone, but had fewer side effects. ^1^ Since then, physicians outside the U.S. have prescribed it off-label for DMD, and some American patients have reportedly obtained it from abroad, as permitted under FDA’s personal importation policies. ^a^ Marathon Pharmaceuticals purchased the rights to deflazacort, completed the data analysis ^2^, and received FDA approval for the drug (now called Emflaza) in February 2017. ^b^

Marathon’s announcement that it intended to raise Emflaza’s U.S. price 60-fold compared with its price in the U.K. attracted substantial scrutiny and censure. Experts estimate Marathon spent about $50M, and possibly far less, to acquire deflazacort and conduct limited additional research. In return for that relatively meager investment, the company was rewarded with seven years of market exclusivity under the Orphan Drug Act for a drug that could earn over $400M annually.

Superficially, Emflaza invites comparison with other recent examples of exorbitant drug price increases taken by companies that invested little to nothing in the therapy’s development. For example, in 2013, Marathon itself acquired two FDA-approved cardiac therapies, Nitropress (sodium nitroprusside) and Isuprel (isoproterenol), from Hospira, then increased the prices about 4-fold before selling the drugs to Valeant in 2015, which immediately further hiked the prices. And in 2015, Turing Pharmaceuticals acquired Daraprim (pyrimethamine), a drug for toxoplasmosis and other infectious diseases, and within months raised the price over 50-fold. In these cases, like with Emflaza, a company reaped outsized returns by substantially increasing the price on a marketed drug that was discovered long ago by another firm.

But Marathon is distinct from these other examples of egregious price increases because before the company gained approval for Emflaza, U.S. physicians could not prescribe deflazacort to DMD patients. By submitting previously unpublished data on the drug for FDA regulatory review, Marathon enabled Americans to easily obtain a clinically useful therapy that was previously inaccessible through normal channels. This is in stark contrast to the examples cited above, as well as other recent cases of excessive price hikes, like cycloserine, Duexis (ibuprofen and famotidine) ^3^, Keveyis (daranide), and Vimovo (esomeprazole and naproxen) – because in all of these other cases, American patients already had unfettered access to the drug (or its active ingredients).

The fact that Emflaza was not previously available by prescription in the U.S. does not itself justify Marathon’s pricing decision, but it highlights a distinctive aspect of this case compared with the other examples cited above. Marathon certainly benefitted from pricing freedom and regulatory advantages that are intended for novel drugs that require substantial at-risk R&D investment, which was not the case with Emflaza. At the same time, however, there is value to enabling American patients to access deflazacort, an effective drug in an under-served disease area, through a standard prescription, without the hassle and expense of acquiring it from abroad. Emflaza illustrates the tension between protecting Americans from predatory pricing behavior on the part of drug manufacturers and encouraging companies to launch meaningful therapies in the U.S. Thus, this particular scenario is not a clear-cut, routine example of pharmaceutical price-gouging, and may warrant specific attention from legislators and regulators.

## Defining the magnitude of the problem

Before considering ways to balance affordability and access in Emflaza-like scenarios, it is worth understanding how common these situations are likely to be in the future. To answer this question, we searched for other “legacy” drugs like deflazacort that are available abroad and have existing off-label data in an orphan indication that might support speedy FDA approval at low cost and risk. ^c^ We focused on orphan drug opportunities because these are most likely to be attractive to biotechs seeking to pursue this strategy: published data from a small trial may suffice for FDA approval, without the need for additional studies; high prices for rare diseases therapies in the U.S. are common, and rarely encounter backlash from payers; and market exclusivity under the Orphan Drug Act makes this strategy feasible for “legacy” therapies.

Using the U.K. as a test case, we identified just one approved drug that we believe could support an Emflaza-like strategy: celiprolol, an off-patent beta-blocker originally developed by Rhône-Poulenc and available cheaply abroad. Published data show that celiprolol prophylaxis significantly reduces arterial rupture and dissection in vascular Ehlers-Danlos syndrome (vEDS), a genetic collagen disorder. ^4^ Thus, we found it highly possible that a firm could obtain the rights to the drug and data, garner FDA approval with little or no additional R&D expense, raise the price substantially over its current U.K. price, and gain orphan drug market exclusivity. In fact, during the preparation of this article we found that a biotech firm, Acer Therapeutics, had already obtained exclusive rights to celiprolol’s vEDS data, and intends to file for FDA approval for the drug (to be called Edsivo) in early 2018, having disclosed no plans for additional clinical trials. Although we analyzed just one non-U.S. country for illustrative purposes, we believe our results suggest that future scenarios directly analogous to Emflaza are possible, but unlikely to be frequent.

## Preventing (or punishing) the next Marathon

Emflaza represents a special case of the general problem of companies taking egregious price increases on old drugs, because it involves a therapy not previously approved by the FDA. Thus, recent state and Federal activities aimed at curbing high prices on off-patent drugs that lack generic competitors or large price increases on already-approved agents will not prevent or punish future Emflaza-like scenarios.

The most effective and least burdensome approach to prevent other firms from copying Marathon’s Emflaza strategy would likely involve alterations to FDA’s existing rules on personal importation. ^d^ Americans are currently permitted to import drugs for non-serious conditions for personal use if they are “not known to represent a significant health risk”. For “serious” conditions, importation of drugs deemed safe is allowed for personal use only if the drug is neither available nor promoted commercially in the U.S. Refining these criteria to allow Americans to import generics in certain situations where a drug with the same active ingredient is already available in the U.S. – for example, if the generic was launched abroad before the first FDA approval – would permit continued importation of drugs like deflazacort and celiprolol, and thus eliminate the incentive for future companies to take a similar approach. At the same time, such a change would not jeopardize the incentives for drug developers to invest in R&D to develop *bona fide* new therapies and bring them to the U.S. market.

Other options to prevent or punish firms pursuing Marathon’s Emflaza strategy are also theoretically possible, but may be more challenging to implement. It is extremely difficult to define criteria that could allow FDA to block drugs like Emflaza while still approving “legitimate” drugs applying for U.S. market access (based, for example, on an (arbitrary) minimum amount of R&D investment), and in the absence of such a definition, providing FDA with broad latitude to refuse approval to drugs like Emflaza on a case-by-case basis – a sort of “I know it when I see it” approach to drug price gamesmanship – is unlikely to withstand legal scrutiny. FDA could also enact policies to automatically grant FDA approval to drugs already available outside the U.S., but versions of such “reciprocal approval” policies proposed to date could have undesirable safety implications for American patients that have not been fully explored. ^5^ And on the pricing side, although there has been significant recent effort in state legislatures to control price increases on drugs already available in the U.S., there has been scant progress on fairly controlling launch prices of new drugs, which is the key issue for Emflaza. ^6^

## Conclusions

Although pharmaceutical industry critics commonly lump together various drug price scandals, there are important differences between them that may require, in some cases, bespoke solutions. Marathon Pharmaceuticals illustrates a very specific challenge for legislators and regulators: the potential for companies to gain FDA approval for old drugs available outside the U.S., often under policies intended to support innovative R&D in rare diseases, and then take advantage of the permissive American drug pricing environment to substantially raise their prices. This situation, though likely to be rare in the future, will not be prevented by most of the drug pricing policies currently under consideration at the state and Federal level, and thus may warrant focused attention and a customized approach. Among several potential solutions, a change to FDA’s personal importation policies is most likely to prevent future Emflaza-like scenarios while having little to no impact on the broader U.S. system that rewards drug companies for investing in risky, expensive R&D in clinically important new therapies.

## Footnotes

a
U.S. FDA Regulatory Procedures Manual, Ch. 9: Import Operations and Actions.

b The company subsequently sold the rights to another biotech company, PTC Therapeutics.

c On February 18, 2017, we downloaded complete lists of drugs approved in the U.S. (2,610) and U.K. (1,879) from https://www.fda.gov/Drugs/InformationOnDrugs/ucm079750.htm and https://www.medicines.org.uk/emc/browse-ingredients, respectively. We used text matching and manual curation to identify active ingredients available only in the U.K., eliminating those that were already approved or generally recognized as safe by FDA (and alternate salt forms thereof) or available over the counter in the U.K. We also eliminated various non-therapeutic agents, including diagnostics, cosmetics, food additives, allergy testing antigens, and herbal and nutritional agents (including oils and vitamins). Finally, we excluded vaccines, biologics, cancer therapies, and any drug under active development by its originating company. For the remaining 133 drugs (see Supplementary File 1), we searched clinicaltrials.gov and PubMed for positive efficacy data in a new (currently off-label) orphan indication.

d
U.S. FDA Regulatory Procedures Manual, Ch. 9: Import Operations and Actions.

## Supplementary material

**Supplementary File 1: Potential future “Emflaza-like” opportunities subjected to further analysis.** Active ingredients available by prescription in the U.K., but not the U.S., on February 18, 2017, that we analyzed manually (N=133). See text for details.

## References

[1] Brooke MH: A randomized trial of deflazacort and prednisone in Duchenne muscular dystrophy: efficacy and toxicity [abstract]. *Neurology.* 1996;46:A476 Reference Source

[2] Griggs RC Miller JP Greenberg CR: Efficacy and safety of deflazacort vs prednisone and placebo for Duchenne muscular dystrophy. *Neurology.* 2016;87(20):2123–2131. 10.1212/WNL.0000000000003217 27566742 PMC5109941

[3] Hakim A Ross JS: High Prices for Drugs With Generic Alternatives: The Curious Case of Duexis. *JAMA Intern Med.* 2017;177(3):305–306. 10.1001/jamainternmed.2016.8423 28114637

[4] Ong KT Perdu J De Backer J: Effect of celiprolol on prevention of cardiovascular events in vascular Ehlers-Danlos syndrome: a prospective randomised, open, blinded-endpoints trial. *Lancet.* 2010;376(9751):1476–1484. 10.1016/S0140-6736(10)60960-9 20825986

[5] Larochelle M Downing NS Ross JS: Assessing the potential clinical impact of reciprocal drug approval legislation on access to novel therapeutics in the USA: a cohort study. *BMJ Open.* 2017;7(2):e014582. 10.1136/bmjopen-2016-014582 28179418 PMC5306516

[6] Berman A Lee T Pan A: Curbing unfair drug prices: A primer for states. Yale Global Health Justice Partnership, August2017 Reference Source

