# Supplementary material for: How to prevent the next Marathon Pharmaceuticals
Source: F1000Res. 2018 Jan 17;7:74. [Version 1] doi: 10.12688/f1000research.13678.1 (PMC5865197; doi:10.12688/f1000research.13678.1)
Supplement: Peer review history

# Peer review: Referee response for version 1

**DOI:** 10.5256/f1000research.14858.r32159

**Published:** 2018-03-22

**Authors:** John L. LaMattina

**Affiliations:** 1 PureTech Health, Boston, MA, USA

  The authors discuss an important issue regarding the pricing of drugs. The issue is presented clearly and concisely, and is put in the context of other drug pricing situations where egregious prices are being charges. The authors then propose a solution to prevent such an example from occurring in the future.

       It is important for this paper to be indexed as it will spark a debate about how to prevent a company like Marathon from exploiting loopholes in the US orphan drug approvals process.The Emflaza case is one where a company is charging far too high a price for an important DMD drug which has been available for decades in Europe for far less money. Furthermore, the authors show that this is not a unique situation and that it is likely to be repeated by another company. Thus, a solution is needed.

        The authors propose a potential solution which should be aired and debated. Thus, I fully endorse indexing of this paper.

       Having said that, I am not fully on board with the authors' solution. Marathon DID spend $50 million on clinical studies to prove the medical viability of Emflaza in DMD patients. Yes, the drug is being used off-label in Europe for DMD, but have studies been published justifying its use? If  yes, how vigorous are these studies? Depending on the answers to these questions, one could argue that Marathon did a service for physicians and patients by carrying out a phase 3 program that was of sufficient rigor to garner FDA approval. 

       The authors' solution could work had the NIH carried out these studies and proved the value of Emplaza, thereby justifying the decision to allow Americans to import generics. 

       Again, my questions only serve to justify indexing of this paper to spur such debate.

I have read this submission. I believe that I have an appropriate level of expertise to confirm that it is of an acceptable scientific standard.

# Peer review: Referee response for version 1

**DOI:** 10.5256/f1000research.14858.r30139

**Published:** 2018-02-21

**Authors:** Rena M. Conti

**Affiliations:** 1 Department of Public Health Sciences, The University of Chicago, Chicago, IL, USA

On the topic of high drug pricing in the US, the authors present an interesting case and thoughtfully discuss it's potential implications.

 I appreciate their attempt to quantify the prevalence of future potential cases, but felt this effort could have been more systematic and involved other OECD countries.  

 On motivation, I think it would have been preferable article construction to start with the problem of the pricing of new drugs in the US, particularly orphans, and then located this as a "special" case.  

 Generally, the authors could have better described the tradeoffs presented in this case regarding access - entry facilitates wider access to a drug that may help American patients suffering from disease, but also creates access and affordability concerns due to the aggressive pricing practices of it's manufacturer.  

 Within this context, the authors insight that this case highlights the special role the FDA may play in altering pricing incentives drug manufacturers face in the US is good and particularly helpful.

 Some of the terminology used in the article to discuss the current political debate regarding high prices - notably the public pursuit of "punishing" manufacturers for high prices - is a bit too dismissive and could be revised. Besides from naming and shaming through the press and Congressional hearings, there has been little "punishment" doled out to manufacturers that pursue such aggressive pricing behavior.  What punishment exists is related to pricing practices that are specifically illegal as defined by laws and statutes. The main point here should be that the manufacturer's aggressive pricing practices may hurt American patients' access to care and presents real tradeoffs to payers; what to do about this, if anything, is a real concern for patients, their families and policymakers.

I have read this submission. I believe that I have an appropriate level of expertise to confirm that it is of an acceptable scientific standard, however I have significant reservations, as outlined above.

# Peer review: Referee response for version 1

**DOI:** 10.5256/f1000research.14858.r30142

**Published:** 2018-01-24

**Authors:** Alexander Tabarrok

**Affiliations:** 1 Department of Economics, George Mason University, Fairfax, VA, USA

Massive increases in the prices of some drugs have generated significant outrage and media attention. Most infamously, Turing Pharmaceutical's increase in the price of Daraprim from $13.50 to $750 made headlines around the world. In part, no doubt, because the antics of Turing CEO Martin Shkreli made him an impossible to ignore villain straight out of central casting.

 In this paper, David and Dixit look at a seemingly similar case, Marathon Pharmaceutical's decision to price Emflaza at $89,000 a year when it is available elsewhere in the world at a price on the order of $1500 a year. David and Dixit caution that the two cases need to be distinguished because prior to Marathon, Emfalza was not for sale in the United States at any price. Marathon performed a useful service in seeking and obtaining FDA approval and there has to be a balancing of costs and benefits if we want to continue to incentivize firms to look for other pharmaceuticals from around the world and make them available in the United States.

 I agree with David and Dixit's analysis but I would go further. While David and Dixit suggest that we need "bespoke solutions" I worry that in trying to solve every problem we will create a mass of regulations that raise costs and harm American patients overall. We need to look beyond the headlines at the bigger picture.

 Although a handful of generics have seen extreme increases in prices, the prices of generics overall have been falling. The Department of Health and Human Services in 2016 concluded, for example, that big price increases were confined to small markets and that overall total spending and prices were falling.

 https://aspe.hhs.gov/pdf-report/understanding-recent-trends-generic-drug-prices

 More references here:

 http://marginalrevolution.com/marginalrevolution/2016/02/the-good-news-on-generic-drugs.html

 The baby in this case is much bigger and more valuable than the bathwater so even though massive increase in prices feel unjust and unfair we need to be extra careful not to throw the baby out with the bathwater. The rules governing the US generic markets are working very well. US prices for generics are low by world standards and availability is high.

 Branded pharmaceuticals in the United States are, of course, very expensive. But what needs to be stressed is that by a time a drug is approved, patents last only about 10-15 years and then prices fall rapidly with generic competition. Moreover, a large majority of prescriptions are for generics. Even more importantly, it's vital that we continue to produce new drugs to reduce morbidity and mortality. In any welfare comparison, high prices are a small price to pay for better life-saving and live-improving medicines.

 Thus, I agree with the David and Dixit analysis but suggest that broadening the picture would strengthen their argument even further.

I have read this submission. I believe that I have an appropriate level of expertise to confirm that it is of an acceptable scientific standard.
